# An Overview of Live Attenuated Recombinant Pseudorabies Viruses for Use as Novel Vaccines

**DOI:** 10.1155/2014/824630

**Published:** 2014-06-05

**Authors:** Bo Dong, Dante S. Zarlenga, Xiaofeng Ren

**Affiliations:** ^1^Department of Preventive Veterinary Medicine, College of Veterinary Medicine, Northeast Agricultural University, 59 Mucai Street, Xiangfang District, Harbin 150030, China; ^2^Animal Parasitic Diseases Laboratory, Agricultural Research Service, Beltsville, MD 20705, USA

## Abstract

Pseudorabies virus (PRV) is a double-stranded, DNA-based swine virus with a genome approximating 150 kb in size. PRV has many nonessential genes which can be replaced with genes encoding heterologous antigens but without deleterious effects on virus propagation. Recombinant PRVs expressing both native and foreign antigens are able to stimulate immune responses. In this paper, we review the current status of live attenuated recombinant PRVs and live PRV-based vector vaccines with potential for controlling viral infections in animals.

## 1. Introduction

### 1.1. Background Information on PRV

Pseudorabies virus (PRV) is a member of the family Herpesviridae, subfamily Alphaherpesvirinae [1] and the causative agent of pseudorabies (PR) or Aujezsky's disease. Infections with PRV result in nervous disorders, respiratory distress, weight loss, young piglet death, and abortion [2]. The virus has a double-stranded linear DNA genome 1.43 × 10^5^ kb in length [3] and contains a unique long region (UL), a unique short region (US), a terminal repeat sequence (TRS), and internal repeat sequences (IRS) [4].

**Figure 1:**
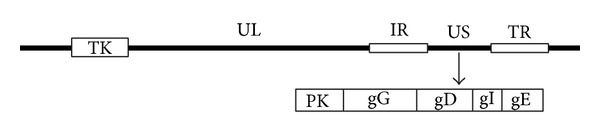
Common sites in the PRV genome for inserting exogenous genes. The genes encoding TK, PK, gG, gD, gI, and gE are the most common sites for inserting exogenous sequences. The TK gene is located within the unique long region (UL), and the PK, gG, gD, gI and gE genes are located within the unique short region (US). IR = internal repeat sequences; TR = terminal repeat sequence. The drawing is not to scale.

To date, at least 11 different glycoproteins of PRV (gB, gC, gD, gE, gG, gH, gI, gK, gL, gM, or gN) have been identified and the genes that encode these proteins have been sequenced. The essential glycoproteins of PRV include gB gD, gH, gL, and gK; the others are considered nonessential [5, 6]. There are several nonstructural proteins of PRV such as thymus kinase (TK) and protein kinase (PK), which are associated with virulence [6, 7]; however, this subset of genes can be replaced by heterologous genes without affecting infectivity or virus propagation provided the essential genes remain intact. A schematic drawing regarding common sites for gene insertion in the PRV genome is shown in Figure 1.

The efficacy of multivalent PRV vaccines has been investigated. Herein, we review research progress using attenuated recombinant PRVs (rPRVs) as vaccine candidates with application for advancing the development of rPRV vector vaccines.

### 1.2. Introduction to Live Attenuated PRV Vaccines

Recombinant viruses represent a particularly promising avenue of vaccine research both for improving existing vaccines and for developing new ones [8, 9]. In principle, the design of PRV vector vaccines is predicated upon the genome of live PRV being used to insert and express genes encoding protective antigens from other pathogens including viruses, bacteria, and parasites [10]. The expressed foreign antigens can be used subsequently to stimulate relevant immune responses [11]. The existence of numerous nonessential genes in the large PRV genome permits the simultaneous insertion of multiple foreign genes in the hope of vaccinating against several diseases at the same time [12].

PRV Bartha-K61 is a common parental strain of rPRV. It is an attenuated PRV which has been passaged repeatedly in pig kidney cells, chicken eggs, and chicken embryo cells [13]. In this strain, the complete gE and part of gI genes have been deleted [14]. Nonetheless, this construct has met with good success in developing multivalent vaccines to control various infectious diseases [4, 10, 15].

The common strategy for using rPRV involves constructing a transfer vector harboring a portion of the PRV genome. This vector is transfected into susceptible cells along with the native PRV, and then the cells are screened for the presence of the recombinant. In addition to a portion of the PRV genome, the transfer vector also contains a promoter, the foreign genes of interest, and a reporter gene. PRV sequences should appear at the start and end of the vector to permit homologous recombination between the arms of the vector and virus genomes. One study demonstrated that the human cytomegalovirus (CMV) promoter is more efficient than the PRV promotor in directing viral gene synthesis [4]. As such, the immediate early gene promoter of CMV has become the most common promoter used in these constructs. It can also be used for identification of rPRVs. In addition to conventional approaches to generating recombinants, viral genomes can be cloned into bacterial artificial chromosome (BAC) vectors. The use of herpesvirus BACs for generating site-directed and transposon mutagenic recombinants has been reviewed [16].

## 2. The Efficacy of Live Attenuated PRV Vaccines

To date, most of the foreign genes that have been inserted into the PRV genome encode key antigens derived from animal viruses. A summary of constructs developed to date is provided in Table 1 which includes parental PRV strains, foreign genes, and insertion sites. The examples which follow provide a more in-depth discussion of successes using this technology.

### 2.1. PRRSV/PRV Recombinant Virus Vaccines

Porcine reproductive and respiratory syndrome virus (PRRSV) is an enveloped, positive strand RNA virus which is a member of the familyArteriviridae [17]. It causes tremendous economic losses worldwide and is among the most important diseases in countries where swine are intensively raised [18–20]. The genome of PRRSV is 15 kb in length and contains nine open reading frames (ORFs) designated ORF1a, ORF1b, ORF2a, ORF2b,ORF3, ORF4, ORF5, ORF6, and ORF7 [21–23].

A decade ago, an attenuated rPRV, rPRV-GP5, was developed that expresses the GP5 envelope protein of PRRSV; the GP5 protein is encoded by ORF5. The rPRV-GP5 was able to confer significant protection against clinical symptoms and reduce pathogenic lesions caused by PRRSV challenge in vaccinated pigs. Pigs immunized either with rPRV or with PRRSV inactivated vaccine remained clinically healthy before and after challenge. Following immunization, only a short period (3 days) of mild fever (≤41°C), gradually improving lung and kidney lesions, and short-term viremia (2 and 3 weeks, resp.) resulted; however, no anti-PRRSV antibody was detected before challenge [24]. In order to improve the protective efficacy of rPRV-GP5, a modified GP5 gene (GP5 m) was synthesized wherein a Pan DR T-helper cell epitope (PADRE) sequence was inserted between the N-terminus and the neutralizing GP5 epitope. The new rPRV-GP5 m elicited a higher level of PRRSV-specific neutralizing antibodies and cellular immune responses than the rPRV-GP5 [25].

Recently, this group generated another construct named rPRV-GP5 m-M that expresses two major membrane-associated proteins (GP5 and M) of PRRSV within the same vector [11]. Mice immunized with rPRV-GP5 m-M developed PRV-specific humoral immune responses and provided complete protection against a lethal PRV challenge. At the same time, high levels of PRRSV-specific neutralizing antibodies and lymphocyte proliferation responses were observed in the immunized mice. Once proof of principle was demonstrated in mice, studies advanced to piglets. When compared to the commercially available PRRSV killed vaccine, rPRV-GP5 m-M immunized animals generated higher PRRSV-specific neutralizing antibodies and higher lymphocyte proliferation responses resulting in better protection against PRRSV. These data indicate that PRV is an excellent vector for developing virus-based vaccines against PRV and PRRSV.

### 2.2. PCV2/PRV Recombinant Virus Vaccine

Porcine circovirus type 2 (PCV2) is the primary cause of postweaning multisystemic wasting syndrome (PMWS), which is a worldwide disease that debilitates pigs with lymphadenopathy and interstitial pneumonia [26, 27]. PCV2 is a single-stranded circular DNA virus and a member of the family Circoviridae [28]. PCV2 has three major ORFs; ORF1 encodes two replication-related proteins, Rep and Rep', which are essential for viral DNA replication [29]; ORF2 encodes the major capsid protein of PCV2; and ORF3 encodes the nonstructural ORF3 protein [30]. A rPRV expressing a fusion protein of ORF1 and 2 was constructed and its immunogenicity tested in mice and pigs [10]. The rPRV-PCV2 elicited strong anti-PRV and anti-PCV2 antibodies in BALB/c mice wherein rPRV-PCV2 protected mice against a lethal challenge with a virulent PRV. In pigs, rPRV-PCV2 elicited significant immune responses against PRV and PCV2.

A second rPRV was constructed expressing only the ORF2 gene that was also used to immunize piglets. Results showed that the rPRV-ORF2 elicited significant humoral immune responses to both PRV and PCV2 wherein PCV2-specific lymphocyte proliferation responses could be detected by 49 days after immunization [31]. The rPRV-ORF2 was better at eliciting protective immune responses in piglets than rPRV expressing both ORF1 and 2. These findings demonstrate that rPRV-PCV2 may be a suitable bivalent vaccine against PRV and PCV2 and that multiplicity is not always the optimal approach to vaccine development.

### 2.3. FMDV/PRV Recombinant Virus Vaccine

Foot-and-mouth disease virus (FMDV) is highly contagious and affects all cloven-hoofed domestic animals including cattle, sheep, goats, pigs, and buffalo [32]. It is a positive single-stranded RNA virus approximately 8.5kb in length and belongs to the family Picornaviridae. The FMDV capsid precursor P1-2A is cleaved and released from the polyprotein by L protease and processed by viral protease 3C to form four structural proteins, VP1, VP2, VP3, and VP4 [33]. FMDV has seven serotypes, A, O, C, Asia1, SAT1, SAT2, and SAT3, each of which contains multiple subtypes [34–37].

Using PRV as a vector, the VP1 gene was fused to the PRV genome. The immunogenicity of the recombinant product was tested in 15 FMDV seronegative white pigs. Although the antibody levels were lower than those induced by commercially available FMDV vaccines and protection against virulent FMDV was not observed, the rPRV-VP1 construct still alleviated clinical symptoms in infected pigs [38]. To improve the immune response, another rPRV was generated that expresses P1-2A of FMDV; its protective effects were evaluated in white pigs [39]. In contrast to the earlier version, these pigs exhibited high levels of neutralizing antibodies to both FMDV and PRV and further showed strong CTL responses against FMDV antigen activation. Following challenge, replication of FMDV was significantly lower in pigs vaccinated with the new rPRV construct when compared to the commercially available vaccine.

Recently, another rPRV which coexpresses P1-2A and the viral protease 3C was developed and tested in piglets [40]. These results showed that rPRV-P12A3C induced a high level of neutralizing antibodies and FMDV-specific lymphocyte proliferative responses. Relative to the inactivated FMDV vaccine which provided 100% protection, the rPRV-P12A3C induced only 60% protection in challenged piglets but was able to reduce pathogenic lesions. These findings suggest that rPRV-P12A3C was better at protecting piglets than the previous constructs and support further development of vaccines against both FMDV and PRV. Work must now focus on targeting other serotypes of FMDV with the hope of finding one vaccine with good efficacy against all or most serotypes.

### 2.4. PPV/PRV Recombinant Virus Vaccine

Porcine parvovirus (PPV) is an important cause of reproductive failure in swine. It is characterized by fetal death, mummification, stillbirth, and prolonged farrowing intervals [41]. PPV is a single-strand DNA virus, which is a member of the familyParvoviridae. Its genome is 5kb is size and contains two large ORFs; the left ORF encodes the nonstructural protein NS1 and the right ORF encodes three capsid proteins [42]. One of the three capsid proteins, VP2, can self-assemble into virus-like particles (VLPs) that are immunologically indistinguishable from inactivated whole-virus vaccines [43].

A rPRV was constructed to express the VP2 gene of PPV [44]. Piglets vaccinated with rPRV-VP2 elicited PRV- and PPV-specific humoral immune responses and generated complete protection against a lethal dose of PRV. This finding lends further support to the development of bivalent vaccines and in particular, against PRV and PPV.

### 2.5. FMDV/PPV/PRV Recombinant Virus Vaccine

A rPRV coexpressing P1-2A of FMDV and VP2 of PPV was constructed and used to vaccinate BALB/c mice [12]. Both total antibody and neutralizing antibody levels to PRV were equivalent to the commercially available PRV vaccine. Protection to FMDV or PPV was >60% when compared to inactivated vaccines. Neutralizing antibody titers induced by the rPRV construct against FMDV or PPV were 50% of the level induced by their respective inactivated vaccines.

Unlike previous constructs, this vaccine candidate demonstrated the feasibility of using rPRV to develop trivalent vaccines, in particular against PRV, FMDV, and PPV. Future work should be performed in swine to test the utility of such vaccine in the natural host for these viruses.

### 2.6. CSFV/PRV Recombinant Virus Vaccine

Classical swine fever virus (CSFV) is a significant impediment to global trade in swine products and results in considerable financial loss [45]. CSFV is an enveloped, positive, single-stranded RNA virus which belongs to the genus* Pestivirus* of the family Flaviviridiae [46, 47]. Its genome, which is 12.3 kb long, encodes a single glycoprotein [48], glycoprotein El (later called E2), which is highly immunogenic and capable of inducing protective immune responses [49, 50].

A bivalent rPRV was synthesized that was gD/gE negative and that expressed glycoprotein E2. Vaccination of piglets exhibited strong protection against both Aujeszky's disease and CSFV [51] supporting the use of rPRV-based bivalent vaccines against CSFV.

### 2.7. SIV/PRV Recombinant Virus Vaccine

Swine influenza virus (SIV) is a type A virus, which is enveloped and consists of negative single-stranded RNA. It is a member of the family Orthomyxoviridaeand its genome encodes 10 viral proteins. RNA segment 4 contains the gene encoding the large hemagglutinin (HA) glycoprotein which is the major surface glycoprotein. It is also a major immunogen which induces subtype-specific protective cellular and humoral immune responses in animals [52, 53]. Segment 5 encodes the nucleoprotein (NP) gene [54]

A rPRV expressing the HA gene of serotype H3N2 subtype SIV (A/Swine/Inner Mongolia/547/2001) was constructed [55] and its immunogenicity was tested in mice. Upon challenge, no virus could be isolated from the vaccinated mice; however, mild pathological lesions were observed in the lungs. At the same time, the rPRV-HA construct protected mice from challenge using a heterologous virulent SIV (A/Swine/Heilongjiang/74/2000) as well. The rPRV-HA vaccine represents a candidate vaccine against SIV. Recently, Klingbeil et al. [56] used BAC technology to generate a HA-based vaccine derived from the swine H1N1 virus cloned into PrV. The resulting virus showed little difference from the parental strain. Pigs given a single injection of the vaccine produced high levels of antibody directed at the H1N1-derived HA protein and were protected from clinical signs of infection when challenged.

### 2.8. Other rPRV Vaccines

PRV has a wide range of hosts including swine, sheep, cattle, and dogs [3]. As such, the PRV vector has been used to develop recombinant vaccines in other hosts and in systems unrelated to viral protection, that is, protozoan parasites.

#### 2.8.1. *Toxoplasma gondii*/PRV Recombinants

A rPRV was constructed expressing SAG1 from the protozoan parasite,* T. gondii* [57]. The SAG1 protein domain belongs to a group of glycosylphosphatidylinositol (GPI)-linked proteins with SAG1 related sequences that can be found on the surface of the parasite. The protective character of the rPRV-SAG1 construct was tested in BALB/c mice. All mice vaccinated with the rPRV-SAG1 developed high levels of specific antibodies against* T. gondii* lysate antigen (TLA) and neutralizing antibodies. In addition, they observed an increase in the splenocyte proliferative response, IFN-*γ* and IL-2 and strong cytotoxic T lymphocyte responses. When the mice were challenged with the highly virulent RH strain of* T. gondii*, the rPRV-SAG1 construct induced partial protection (60%). This is likely related to the significantly complex life cycle of protozoan parasites and the stage specificity of SAG1 expression.

In order to improve the protective response, two additional rPRVs expressing SAG1 or the micronemal protein MIC3 (rPRV-MIC3) were developed and used to immunize BALB/c mice separately and simultaneously [58]. All mice vaccinated with the rPRVs induced high levels of antibodies to* T. gondii* lysate antigen, splenocyte proliferation, IFN-*γ*, and IL-2. Further experiments indicated that rPRVs stimulated humoral and cellular immune responses in vivo. The vaccinated mice survived a lethal challenge with* T. gondii* RH strain; however, protection was not complete.

These results support previous studies showing the utility of expressing* T. gondii* protective antigens in PRV as a novel approach for developing vaccine candidates against pseudorabies and toxoplasmosis; however additional research is needed to increase the survivability of host animals to parasite challenge. One approach is to make a multivalent vaccine that targets more than one stage of infection or to test other parasite antigens. Unlike viruses, parasites are far more complex both biologically and genetically which complicates the approach to recombinant vaccine development.

#### 2.8.2. *Schistosoma japonicum*/PRV Recombinants

Three rPRVs expressing* S. japonicum* glutathione S-transferase (Sj26GST), fatty acid binding protein (SjFABP), or both were constructed and named rPRV/Sj26GST, rPRV/SjFABP, and rPRV/Sj26GST-SjFABP, respectively [59]. Their abilities to protect mice and sheep against* S. japonicum* challenge were evaluated. The results showed that all rPRVs induced specific antibody responses against total worm extracts, increased splenocyte proliferation, and elevated IFN-*γ* and IL-2 levels in the immunized mice. However, better immune stimulation was observed in animals given rPRV/Sj26GST-SjFABP than in those given either rPRV/Sj26GST or rPRV/SjFABP. Further, in all immunized sheep, the treatment was deemed safe and the worm and egg burdens were demonstrably reduced following challenge.

These results indicated that the multivalent rPRV-based vaccines for* S. japonicum* can produce significant protection and are capable of preventing infection from protozoan parasites. However, less than 100% protection, which is very common among putative parasite vaccines, has hindered acceptance and further development.

#### 2.8.3. JEV/PRV Recombinants

A rPRV expressing the NS1 protein of Japanese encephalitis virus (JEV) was constructed [60]. Both BALB/c mice and pigs were immunized. A test using 10^6^ pfu, in mice, piglets, and pregnant sows indicated a good safety profile for the rPRV. Animals given the rPRV-NS1 virus developed JEV-specific humoral and cellular immune responses and protected the animals from a lethal challenge with the virulent PRV Ea strain. These experiments provided evidence that the rPRV may serve as a candidate for generating a novel vaccine that can be used for controlling pseudorabies and Japanese encephalitis.

#### 2.8.4. Rabies Virus/PRV Recombinants

A rPRV expressing the rabies virus glycoprotein was constructed [61]. This recombinant virus was deemed safe for dogs by oral and intramuscular inoculation routes and induced protective immune responses against both rabies and pseudorabies. Neutralizing antibody titers against rabies and pseudorabies were demonstrably elevated by 5 weeks after vaccination and remained as such for at least 6 months. This experiment indicates that constructs designed herein survived well in the host such that the immune profile of vaccinated animals was long-lived.

## 3. Other Virus Vectors

Clearly, there are other viral genomes that can serve as vaccine vectors such as adenovirus, poxvirus, and baculovirus. These have all been tested as delivery vehicles for exogenous antigens that had been previously expressed in PRV vectors. Adenoviruses are currently one of the most applied systems for gene delivery. As vectors, they have a high capacity for the insertion of foreign genes (5–36 kb), are able to transduce a broad range of cell types [62], and are commercially available in kit form for subsequent genetic modification.

Poxviruses are the largest known group of animal DNA viruses. They have been extensively used as expression vectors for vaccination, expression of large foreign genes, and induction of cellular and humoral immune responses [63]. Among the more common poxviruses are modified vaccinia virus Ankara (MVA), fowlpox virus, and orf virus. MVA has been a smallpox vaccine for many years and more recently it has been used as a viral vector for preventing both cancer and infectious diseases [64]. Other examples include the use of a canarypox-based recombinant containing the PrM and E genes of the West Nile Virus (WNV) to induce protection in cats and dogs. This study resulted in the expression of the WNV genes and the induction of protective immunity [65]. The orf virus has been used to generate protective immunity against CSV using the E2 gene [66] and pseudorabies in pigs [67]. Inasmuch as the orf virus rarely causes system infections and has a narrow infection host range; it is a logical choice for developing multivalent viral vaccines.

Baculovirus is an excellent tool to overexpress recombinant proteins in insect cells. Its host specificity was originally thought to be restricted to cells derived from arthropods; however, recent studies have shown that baculoviruses carrying mammalian cell-active promoters are capable of transferring and expressing foreign genes in a variety of mammalian cell types as well as in animal models [68]. Baculovirus systems have been very popular because like adenoviruses, they also are available commercially and in kit form for easy genetic modification.

Above-mentioned viruses have also been used to develop recombinant live viruses bearing components of PRRSV, PCV2, FMDV, CSFV, and SIV. A comparison of key immunological efficacies among these many virus vectors is shown in Table 2.

## 4. Concluding Remarks

Past successes of rPRV as a vector for expressing exogenous antigens has resulted in new rPRVs being constructed that are less pathogenic. There are many advantages of rPRV. First, live attenuated PRV has a large genome wherein half of the genome is considered nonessential thus permitting modification without affecting key characters such as infectivity. Although some of these genes are associated with virulence, their deletion and replacement by foreign genes has no adverse effects on the propagation of PRV [31]. Representative information regarding common insertion sites in parental viruses is summarized in Table 1. The benefits of viral vectors are that they not only express their own protective antigens, but any inserted exogenous genes as well. Inasmuch as they use host machinery to replicate and express proteins, the resultant exogenous gene products have a higher probability of being correctly modified or folded posttranslationally, something which is lacking in bacterial systems. As such, products derived from rPRVs are more likely to mimic native immunogens and correctly induce humoral and/or cellular responses in immunized animals. As shown above, one can target multiple diseases within a single vector construct. Second, there is minimal risk using PRV gene-deletion vaccines. PRV vaccine strains have been used for decades and exhibit high safety and efficacy profiles in vivo. Third, PRV has a broad host range including pigs, cattle, goats, and dogs among others. This makes it possible to target animal diseases in multiple hosts without resorting to multiple vector constructs to express the antigens. Fourth, native PRV induces cellular immunity and causes latent infection. Therefore, rPRVs can be maintained for long periods in a given host thereby providing constant stimulation of the protective immune responses. Finally, PRV can be propagated in various cell lines including SPF chicken embryo fibroblast cells. This permits simplifying virus production and keeping manufacturing costs under control.

Other points to consider when developing PRV-based vector vaccines are that this vector system requires a strong promoter to maintain high and stable expression levels. Also, selection of nonessential genes in the PRV genome to be replaced with the foreign genes of interest can affect optimizing the immune response. Given competing interests between vector-derived and exogenous protein-derived immune responses, recombinant constructs should be characterized with respect to optimal inoculation dosage. For development of effective rPRV vaccines, the pathogenic features, protective mechanisms, and the epidemiology of diseases must be taken into account in all future work.

Many of the rPRV vaccine candidates that have been reported here either have not been further pursued or are not yet commercially available. In general, there are factors that complicate advancing these products to the marketplace. First, optimizing viral infection and replication are required to produce efficient and safe vaccines suitable for release into the environment. To this end, identifying more appropriate nonessential regions within the virus is needed to enhance expression of exogenous genes particularly when multivalent rPRVs are being developed. This is not a trivial task in view of the large genome size of PRV and the interplay between essential regions and exogenous genes that can affect viral virulence and replication. Second, modifications to the parent PRV in generating a rPRV are often required to eliminate or replace existing marker genes or important regulatory elements to make the construct more suitable for clinical application. Third, plans are needed to transition between available vaccinations and those derived from rPRVs. Concurrent or overlapping vaccinations of the two will have a significant and deleterious impact on the efficacy and propagation of subsequent rPRV-based immunizations. Finally, many of the studies using rPRVs have not been advanced to the natural host, that is, swine. Problems with the high cost of clinical trials, manufacturing sufficient amounts to advance these studies and releasing biologicals into the environment are often limiting factors. Yet these studies are necessary to get a more comprehensive picture of the immunogenicity of the expressed genes, the persistence of the viral infection, and longevity of the stimulation in the natural host and to study the potential for tumorigenesis when using uniquely modified rPRV-based vectors.

## Figures and Tables

**Table 1:** General information of recombinant PRVs.

Insertion sites in PRV genome	Parental PRV strains	Foreign Genes [references]
TK gene	Bartha-K61 strain	GP5 of PRRSV [24]
TK gene	Bartha-K61 strain	The HA gene of H3N2 subtype SIV [55]
Between PK and gG gene	Bartha-K61 strain	The major immunodominant surface antigen 1 (TgSAG1) of the protozoan parasite, *T. gondii* [57]
Between PK and gG gene	Bartha-K61 strain	The glycoprotein of rabies virus [61]
Between PK and gG gene	Bartha-K61 strain	The glutathione S-transferase (Sj26GST) and the fatty acid binding protein (SjFABP) of *Schistosoma japonicum* [59]
gG gene	TK-/gG-/LacZ + strain	The VP1 gene of FMDV [38]
gG gene	TK-/gG-/LacZ + strain	The capsid precursor encoding regions of [39]
gG gene	TK-/gG-/EGFP + strain	The main surface antigen 1 (SAG1) and the micronemal protein MIC3 of the protozoan parasite, *T. Gondii* [58]
gG gene	TK-/gG-/LacZ + strain	The NS1 gene of Japanese encephalitis virus [60]
gD gene	gE-/gD-strain	The envelope glycoprotein E2 of CSFV [51]
gI gene	TK-/gE-/gI-strain	The VP2 gene [44]
Between gE and gI gene	TK-/gE-/gI-/LacZ + strain	Two major membrane-associated proteins (GP5 and/or M) (GP5 contains a native GP5 and a modified GP5) of PRRSV [11]
Between gE and gI gene	TK-/gE-/LacZ + strain	ORF1 and partial ORF2 gene/ ORF2 gene of PCV2 [10, 31]
Between gE and gI gene	TK-/gE-/gI-strain	The capsid precursor polypeptide P12A and nonstructural protein 3C of FMDV [40]
Between gE and gI gene	TK-/gE-/LacZ + strain	The protein precursor P1-2A of FMDV and VP2 protein of PPV [12]
gE gene	TK-/gE-/LacZ + strain	The modified GP5 [25]

**Table 2:** Comparisons of immunological efficacies among different virus vectors.

Viruses and vectors	Inserted gene	Host	Neutralizing Ab		Other responses	Reference
*PRRSV *						
Canine adenovirus type 2	GP5 and M	Mouse	Appeared at 14 days post immunization (dpi) peaked at 42 dpi maximum titer = 16		Anti-PRRSV Ab appeared at 14 dpi; CTL appeared at 28 dpi	[7]
Adenovirus	GP5 and M	Mouse	Appeared at 14 dpi peaked at 56 dpi, maximum titer = 102		Specific lymphocyte proliferation responses appeared at 28 dpi; CTL appeared at 28 dpi	[69]
MVA (Poxvirus)	GP5 and M	Mouse	Appeared at 14 dpi peaked at 70 dpi maximum titer = 8.12		High IFN-*γ* (72.6 pg/mL)	[60]
Baculovirus	GP5 and M	Mouse	Appeared at 21 dpi peaked at 42 dpi maximum titer = 8		High IFN-*γ* (147.84 pg/mL)	[52]
PRV	GP5 and M	Mouse	Appeared at 42 dpi peaked at 70 dpi maximum titers = 21.3			[11]
	GP5m and M	Piglets	Appeared at 42 dpi peaked at 84 dpi maximum titer = 160		Anti-PRRSV Ab appeared at 28 dpi	

*PCV2 *						
Adenovirus	ORF2	Piglets	Titers = 1 : 36 (27 dpi) and 1 : 48 (37 dpi)		Specific Ab appeared at 10 dpi; protection = 60%	[55]
Baculovirus	ORF2	Mouse	Appeared at 21 dpi peaked at 42 dpi maximum titer = 16		Specific Ab appeared at 21 dpi; high IFN-*γ* (286 pg/mL)	[70]
PRV	ORF2	Piglets	Appeared at 21 dpi PCV2 Ab not detected		Specific Ab appeared at 21 dpi; PCV2-specific lymphocyte proliferation appeared at 49 dpi (low)	[71]

*FMDV *						
Adenovirus	whole capsid and non-structural protein 3C	Piglets	non-detected		Protection = 75% low FMDV Ab	[72]
Fowlpox virus (Poxvirus)	whole capsid and non-structural protein 3C	Mouse			Specific Ab appeared at 10 dpi	
		Piglets	Peaked at 30 dpi decreased by 49 dpi		Specific Ab appeared at 10 dpi; protection = 75%	[21]
Pseudotype baculovirus	whole capsid and non-structural protein 3C	Mouse	Titer = 13 (21 dpi) Titer = 35 (49 dpi)		High IFN-*γ* (1917 pg/mL)	[73]
PRV	whole capsid and non-structural protein 3C	Piglets	Appeared at 21 dpi (variable)		Virus-specific lymphocyte and non-proliferative responses higher than recombinant; protection = 60%	[63]

*CSFV *						
Adenoviruses	E2 glycoprotein	Rabbits	Titer = 13.8 (21 dpi) Titer = 218.8 (35 dpi)			[43]
		Piglets	Antibody level was at 90% inhibition rate		Protection = 40%	
Orf virus (Poxvirus)	E2 glycoprotein	Piglets	Appeared at 21 dpi Titer = 37 (49 dpi)		Protection = 100%	[74]
PRV	E2 glycoprotein	Piglets	Appeared at 42 dpi Titer = 37		Protection = 100%	[6]

*SIV *						
Adenoviruses	HA gene of type H3N2	Mouse	Appeared at 14 dpi		HA inhibiting (HI) Ab appeared at 14 dpi peaked at 35 dpi Titer = 8; Maximum titer = 32 Protection = 83.3%	[31]
PRV	HA gene of type H3N2	Mouse			HI Ab appeared at 21 dpi Peaked at 42 dpi Titer = 2 Maximum titer = 4 Protection = 80%.	[9]

## References

[1] Nauwynck H, Glorieux S, Favoreel H, Pensaert M (2007). Cell biological and molecular characteristics of pseudorabies virus infections in cell cultures and in pigs with emphasis on the respiratory tract. *Veterinary Research*.

[2] Mettenleiter TC (2000). Aujeszky's disease (pseudorabies) virus: the virus and molecular pathogenesis-state of the art, June 1999. *Veterinary Research*.

[3] Klupp BG, Hengartner CJ, Mettenleiter TC, Enquist LW (2004). Complete annotated sequence of the pseudorabies virus genome. *Journal of Virology*.

[4] Hooft van Iddekinge BJL, de Wind N, Wensvoort G, Kimman TG, Gielkens ALJ, Moormann RJM (1996). Comparison of the protective efficacy of recombinant pseudorabies viruses against pseudorabies and classical swine fever in pigs; influence of different promoters on gene expression and on protection. *Vaccine*.

[5] Kopp M, Granzow H, Fuchs W, Klupp B, Mettenleiter TC (2004). Simultaneous deletion of pseudorabies virus tegument protein UL11 and glycoprotein M severely impairs secondary envelopment. *Journal of Virology*.

[6] Olsen LM, Ch’ng TH, Card JP, Enquist LW (2006). Role of pseudorabies virus Us3 protein kinase during neuronal infection. *Journal of Virology*.

[7] Kelly BJ, Fraefel C, Cunningham AL, Diefenbach RJ (2009). Functional roles of the tegument proteins of herpes simplex virus type 1. *Virus Research*.

[8] Osorio Y, Ghiasi H (2005). Recombinant herpes simplex virus type 1 (HSV-1) codelivering interleukin-12p35 as a molecular adjuvant enhances the protective immune response against ocular HSV-1 challenge. *Journal of Virology*.

[9] Souza APD, Haut L, Reyes-Sandoval A, Pinto AR (2005). Recombinant viruses as vaccines against viral diseases. *Brazilian Journal of Medical and Biological Research*.

[10] Ju C, Fan H, Tan Y (2005). Immunogenicity of a recombinant pseudorabies virus expressing ORF1-ORF2 fusion protein of porcine circovirus type 2. *Veterinary Microbiology*.

[11] Jiang Y, Fang L, Xiao S (2007). Immunogenicity and protective efficacy of recombinant pseudorabies virus expressing the two major membrane-associated proteins of porcine reproductive and respiratory syndrome virus. *Vaccine*.

[12] Hong Q, Qian P, Li XM, Yu XL, Chen HC (2007). A recombinant pseudorabies virus co-expressing capsid proteins precursor P1-2A of FMDV and VP2 protein of porcine parvovirus: a trivalent vaccine candidate. *Biotechnology Letters*.

[13] McFerran JB, Dow C (1975). Studies on immunisation of pigs with the Bartha strain of Aujeszky's disease virus. *Research in Veterinary Science*.

[14] Klupp BG, Lomniczi B, Visser N, Fuchs W, Mettenleiter TC (1995). Mutations affecting the UL21 gene contribute to avirulence of pseudorabies virus vaccine strain Bartha. *Virology*.

[15] Lu JQ, Chen HC, Zhao JL, Fang LR, He QG, Xiong F (2004). The development of recombinant pseudorabies virus expressing porcine parvovirus VP2 gene and the study on its biological characters. *Chinese Journal of Virology*.

[16] Warden C, Tang Q, Zhu H (2011). Herpesvirus BACs: past, present, and future. *Journal of Biomedicine and Biotechnology*.

[17] Meulenberg JJM, Hulst MM, De Meijer EJ (1993). Lelystad virus, the causative agent of porcine epidemic abortion and respiratory syndrome (PEARS), is related to LDV and EAV. *Virology*.

[18] Meng XJ (2000). Heterogeneity of porcine reproductive and respiratory syndrome virus: Implications for current vaccine efficacy and future vaccine development. *Veterinary Microbiology*.

[19] Meulenberg JJM (2000). PRRSV, the virus. *Veterinary Research*.

[20] Murtaugh MP, Xiao Z, Zuckermann F (2002). Immunological responses of swine to porcine reproductive and respiratory syndrome virus infection. *Viral Immunology*.

[21] Wu WH, Fang Y, Farwell R (2001). A 10-kDa structural protein of porcine reproductive and respiratory syndrome virus encoded by ORF2b. *Virology*.

[22] Dea S, Gagnon CA, Mardassi H, Pirzadeh B, Rogan D (2000). Current knowledge on the structural proteins of porcine reproductive and respiratory syndrome (PRRS) virus: comparison of the North American and European isolates. *Archives of Virology*.

[23] Meulenberg JJM, Besten APD, de Kluyver EP, Moormann RJM, Schaaper WMM, Wensvoort G (1995). Characterization of proteins encoded by ORFs 2 to 7 of Lelystad virus. *Virology*.

[24] Qiu HJ, Tian ZJ, Tong GZ (2005). Protective immunity induced by a recombinant pseudorabies virus expressing the GP5 of porcine reproductive and respiratory syndrome virus in piglets. *Veterinary Immunology and Immunopathology*.

[25] Jiang YB, Fang LR, Xiao SB, Zhang H, Chen HC (2005). Construction and immunogenicity of recombinant pseudorabies virus expressing the modified GP5m protein of porcine reproduction and respiratory syndrome virus. *Sheng Wu Gong Cheng Xue Bao*.

[26] Allan GM, McNeilly F, Kennedy S (1998). Isolation of porcine circovirus-like viruses from pigs with a wasting disease in the USA and Europe. *Journal of Veterinary Diagnostic Investigation*.

[27] Ellis JA, Bratanich A, Clark EG (2000). Coinfection by porcine circoviruses and porcine parvovirus in pigs with naturally acquired postweaning multisystemic wasting syndrome. *Journal of Veterinary Diagnostic Investigation*.

[28] Todd D, Weston JH, Soike D, Smyth JA (2001). Genome sequence determinations and analyses of novel circoviruses from goose and pigeon. *Virology*.

[29] Mankertz A, Çaliskan R, Hattermann K (2004). Molecular biology of Porcine circovirus: analyses of gene expression and viral replication. *Veterinary Microbiology*.

[30] Hamel AL, Lin LL, Nayar GPS (1998). Nucleotide sequence of porcine circovirus associated with postweaning multisystemic wasting syndrome in pigs. *Journal of Virology*.

[31] Song Y, Jin M, Zhang S (2007). Generation and immunogenicity of a recombinant pseudorabies virus expressing cap protein of porcine circovirus type 2. *Veterinary Microbiology*.

[32] Sáiz M, Núñez JI, Jimenez-Clavero MA, Baranowski E, Sobrino F (2002). Foot-and-mouth disease virus: biology and prospects for disease control. *Microbes and Infection*.

[33] De Felipe P, Hughes LE, Ryan MD, Brown JD (2003). Co-translational, intraribosomal cleavage of polypeptides by the foot-and-mouth disease virus 2A peptide. *Journal of Biological Chemistry*.

[34] Domingo E, Escarmís C, Baranowski E (2003). Evolution of foot-and-mouth disease virus. *Virus Research*.

[35] Grubman MJ, Baxt B (2004). Foot-and-Mouth Disease. *Clinical Microbiology Reviews*.

[36] Mason PW, Grubman MJ, Baxt B (2003). Molecular basis of pathogenesis of FMDV. *Virus Research*.

[37] Knowles NJ, Samuel AR (2003). Molecular epidemiology of foot-and-mouth disease virus. *Virus Research*.

[38] Qian P, Li XM, Jin ML, Peng GQ, Chen HC (2004). An approach to a FMD vaccine based on genetic engineered attenuated pseudorabies virus: one experiment using VP1 gene alone generates an antibody responds on FMD and pseudorabies in swine. *Vaccine*.

[39] Li X, Liu R, Tang H, Jin M, Chen H, Qian P (2008). Induction of protective immunity in swine by immunization with live attenuated recombinant pseudorabies virus expressing the capsid precursor encoding regions of foot-and-mouth disease virus. *Vaccine*.

[40] Zhang K, Huang J, Wang Q (2011). Recombinant pseudorabies virus expressing P12A and 3C of FMDV can partially protect piglets against FMDV challenge. *Research in Veterinary Science*.

[41] Mengeling WL, Lager KM, Vorwald AC (2000). The effect of porcine parvovirus and porcine reproductive and respiratory syndrome virus on porcine reproductive performance. *Animal Reproduction Science*.

[42] Ranz AI, Manclús JJ, Díaz-Aroca E, Casal JI (1989). Porcine parvovirus: DNA Sequence and Genome Organization. *Journal of General Virology*.

[43] Soares RM, Cortez A, Heinemann MB (2003). Genetic variability of porcine parvovirus isolates revealed by analysis of partial sequences of the structural coding gene VP2. *Journal of General Virology*.

[44] Chen Y, Guo W, Xu Z (2011). A novel recombinant pseudorabies virus expressing parvovirus VP2 gene: immunogenicity and protective efficacy in swine. *Virology Journal*.

[45] Ophuis RJ, Morrissy CJ, Boyle DB (2006). Detection and quantitative pathogenesis study of classical swine fever virus using a real time RT-PCR assay. *Journal of Virological Methods*.

[46] Francki RIB, Fauquet CM, Knudson DL, Brown F (1991). *Classification and Nomenclature of Viruses. 5th Report of the International Committee on Taxonomy of Viruses*.

[47] Huang YL, Pang VF, Pan CH (2009). Development of a reverse transcription multiplex real-time PCR for the detection and genotyping of classical swine fever virus. *Journal of Virological Methods*.

[48] Lowings P, Ibata G, Needham J, Paton D (1996). Classical swine fever virus diversity and evolution. *Journal of General Virology*.

[49] Hulst MM, Westra DF, Wensvoort G, Moormann RJM (1993). Glycoprotein E1 of hog cholera virus expressed in insect cells protects swine from hog cholera. *Journal of Virology*.

[50] Wensvoort G, Boonstra J, Bodzinga BG (1990). Immunoaffinity purification and characterization of the envelope protein E1 of hog cholera virus. *Journal of General Virology*.

[51] Peeters B, Bienkowska-Szewczyk K, Hulst M, Gielkens A, Kimman T (1997). Biologically safe, non-transmissible pseudorabies virus vector vaccine protects pigs against both Aujeszky's disease and classical swine fever. *Journal of General Virology*.

[52] Tang M, Harp JA, Wesley RD (2002). Recombinant adenovirus encoding the HA gene from swine H3N2 influenza virus partially protects mice from challenge with heterologous virus: A/HK/1/68 (H3N2). *Archives of Virology*.

[53] Wesley RD, Tang M, Lager KM (2004). Protection of weaned pigs by vaccination with human adenovirus 5 recombinant viruses expressing the hemagglutinin and the nucleoprotein of H3N2 swine influenza virus. *Vaccine*.

[54] Lamb RA, Krug RM, Knipe DM, Howley PM, Griffin DE (2001). Othomyxoviridae: the viruses and their replication. *Fields Virology*.

[55] Tian ZJ, Zhou GH, Zheng BL (2006). A recombinant pseudorabies virus encoding the HA gene from H3N2 subtype swine influenza virus protects mice from virulent challenge. *Veterinary Immunology and Immunopathology*.

[56] Klingbeil K, Lange E, Teifke JP, Mettenleiter TC, Fuchs W (2014). Immunization of pigs with an attenuated pseudorabies virus recombinant expressing the hemagglutinin of pandemic swine origin H1N1 influenza A virus. *Journal of General Virology*.

[57] Liu Q, Gao S, Jiang L (2008). A recombinant pseudorabies virus expressing TgSAG1 protects against challenge with the virulent *Toxoplasma gondii* RH strain and pseudorabies in BALB/c mice. *Microbes and Infection*.

[58] Nie H, Fang R, Xiong BQ (2011). Immunogenicity and protective efficacy of two recombinant pseudorabies viruses expressing *Toxoplasma gondii* SAG1 and MIC3 proteins. *Veterinary Parasitology*.

[59] Wei F, Zhai Y, Jin H (2010). Development and immunogenicity of a recombinant pseudorabies virus expressing Sj26GST and SjFABP from *Schistosoma japonicum*. *Vaccine*.

[60] Xu G, Xu X, Li Z (2004). Construction of recombinant pseudorabies virus expressing NS1 protein of Japanese encephalitis (SA14-14-2) virus and its safety and immunogenicity. *Vaccine*.

[61] Yuan Z, Zhang S, Liu Y (2008). A recombinant pseudorabies virus expressing rabies virus glycoprotein: safety and immunogenicity in dogs. *Vaccine*.

[62] Mascola JR, Sambor A, Beaudry K (2005). Neutralizing antibodies elicited by immunization of monkeys with DNA plasmids and recombinant adenoviral vectors expressing human immunodeficiency virus type 1 proteins. *Journal of Virology*.

[63] Wong YC, Lin LCW, Melo-Silva CR, Smith SA, Tscharke DC (2011). Engineering recombinant poxviruses using a compact GFP-blasticidin resistance fusion gene for selection. *Journal of Virological Methods*.

[64] Slifka MK (2005). The future of smallpox vaccination: is MVA the key?. *Medical Immunology*.

[65] Karaca K, Bowen R, Austgen LE (2005). Recombinant canarypox vectored West Nile virus (WNV) vaccine protects dogs and cats against a mosquito WNV challenge. *Vaccine*.

[66] Voigt H, Merant C, Wienhold D (2007). Efficient priming against classical swine fever with a safe glycoprotein E2 expressing Orf virus recombinant (ORFV VrV-E2). *Vaccine*.

[67] Dory D, Fischer T, Béven V, Cariolet R, Rziha HJ, Jestin A (2006). Prime-boost immunization using DNA vaccine and recombinant Orf virus protects pigs against Pseudorabies virus (Herpes suid 1). *Vaccine*.

[68] Kost TA, Condreay JP, Jarvis DL (2005). Baculovirus as versatile vectors for protein expression in insect and mammalian cells. *Nature Biotechnology*.

[69] Jiang W, Jiang P, Li Y, Tang J, Wang X, Ma S (2006). Recombinant adenovirus expressing GP5 and M fusion proteins of porcine reproductive and respiratory syndrome virus induce both humoral and cell-mediated immune responses in mice. *Veterinary Immunology and Immunopathology*.

[70] Fan H, Pan Y, Fang L (2008). Construction and immunogenicity of recombinant pseudotype baculovirus expressing the capsid protein of porcine circovirus type 2 in mice. *Journal of Virological Methods*.

[71] Shangjin C, Cortey M, Segalés J (2009). Phylogeny and evolution of the NS1 and VP1/VP2 gene sequences from porcine parvovirus. *Virus Research*.

[72] Liu JM, Cai XZ, Lin JJ (2004). Gene cloning, expression and vaccine testing of *Schistosoma japonicum* SjFABP. *Parasite Immunology*.

[73] Cai J, Ma Y, Li J, Yan C, Hu R, Zhang J (2010). Construction and characterization of a recombinant canine adenovirus expressing GP5 and M proteins of porcine reproductive and respiratory syndrome virus. *Journal of Veterinary Medical Science*.

[74] Sun Y, Li HY, Zhang XJ (2011). Comparison of the protective efficacy of recombinant adenoviruses against classical swine fever. *Immunology Letters*.

